# Chest Tube Placement of Secondary Tracheoesophageal Voice Prosthesis: Overcoming Challenging Anatomy in the Laryngectomy Patient

**DOI:** 10.3390/jpm14101021

**Published:** 2024-09-24

**Authors:** Courtney B. Shires, Joseph S. Schertzer, Lauren Ottenstein, Tricia Harris, Merry E. Sebelik

**Affiliations:** 1 West Cancer Center, Germantown, TN 38138, USA; pharris@westclinic.com; 2 Department of Otolaryngology, School of Medicine, Emory University, Atlanta, GA 30322, USA; joseph.schertzer@emory.edu (J.S.S.); lauren.ottenstein@emoryhealthcare.org (L.O.); merry.sebelik@emory.edu (M.E.S.)

**Keywords:** tracheoesophageal puncture, laryngectomy, trismus, flexible transnasal esophagoscopy, chest tube

## Abstract

**Introduction:** Total laryngectomy is used to cure advanced larynx cancer in many patients. The removal of the larynx requires the rehabilitation of the patient’s ability to communicate, and one common method is to place a tracheoesophageal voice prosthesis (TEP) as a secondary procedure after the patient has completed cancer treatment. The traditional technique utilizes a rigid esophagoscope for access, but this can prove difficult in many patients who have kyphosis, scarring of the neck, or trismus. We describe a technique to allow TEP placement in these challenging patients that does not utilize rigid esophagoscopy to access the tracheoesophageal puncture site. **Methods:** For more than 15 years, the senior authors of this study have used this technique in patients in whom traditional methods of TEP with rigid esophagoscope were unsuccessful or not attempted due to the anticipated high probability of failure. The ease of this technique has prompted its use for all patients undergoing secondary TEP placement in their practice. The technique is described in detail in the Methods section below. **Results:** The described method has been successfully utilized to place TEPs in many patients with challenging anatomy. There have been no failed placements, including a patient with severe trismus who was able to have a TEP placed by placing the chest tube and flexible endoscope transnasally. Further, because of precise visualization and ease of the technique, there have been no observed complications of injury to the pharyngoesophageal lumen or creation of a false passage. **Conclusion:** The use of a chest tube and flexible scope allows for the protection of the pharyngoesophageal lumen, precise visualization and placement of the puncture, and avoidance of a false tracheoesophageal passage, all while minimizing the need for extension of the patient’s neck. This has proven ideal for patients suffering the consequences of cancer treatment such as cervical scarring, fibrosis, kyphosis, and trismus.

## 1. Background

Voice restoration after total laryngectomy has been evolving since Billroth successfully performed the first total laryngectomy (TL) for malignancy in 1873. Over four decades, multiple methods have been proposed throughout the literature. Currently, there are three primary means of speech rehabilitation after TL: electrolarynx, esophageal speech, and tracheoesophageal prosthesis (TEP) speech. As patients undergoing speech restoration after TL are incredibly diverse, there is no single ideal method of speech rehabilitation after TL [1].

TEP speech has become the most popular method of speech restoration, as it results in the greatest patient satisfaction and most natural-sounding voice [2]. TEP placement is accomplished by surgically creating a tracheoesophageal fistula and placing a silicone voice prosthesis into the tract to function as a one-way valve that allows pulmonary air into the esophagus when the tracheostoma is occluded. The transferred air is then able to vibrate the pharyngoesophageal mucosa, resulting in voice production [3]. TEP voice rehabilitation can be performed at the time of total laryngectomy for a primary puncture, or at a later time for secondary puncture.

A systematic review and meta-analysis suggested that the outcomes of primary and secondary TEP are quite similar. The timing of the TEP did not significantly affect voice outcomes, though the trend favored greater voice success with a primary puncture. There was no difference in overall complication rate between the two groups; however, a meta-analysis reported pharyngocutaneous fistula to be significantly more prevalent following primary punctures [4]. In contrast, Barauna noted increased TEP complications among patients who underwent primary puncture [5].

The majority of surgeons choose to perform secondary TEP instead of primary TEP in those patients undergoing salvage surgery after radiation with or without chemotherapy, those undergoing pharyngectomy along with TL, and those undergoing free flap reconstruction after TL.

Chakravarty supported a similar voice outcome with primary puncture and secondary TEP [4]. Chone concluded that voice success rate was significantly higher with primary puncture over secondary puncture [6]. Gitomer found that fluency and voice success were similar after primary and secondary puncture, with a mean follow up of 4.7 years [7]. Sinclair found a median time to voice following primary puncture of 56 days versus 200 days for patients who underwent secondary puncture. They also found no difference in overall surgical complication rate between the two punctures, which was consistent in every manuscript relating to their meta-analysis [8].

As pharyngocutaneous fistula is one of the most common complications after TL, its rates and risk factors have been extensively studied. Chakravarty suggested an increased risk with primary TEP [4]. Emerick also found an increased risk of fistula after TEP in salvage laryngectomy [9]. Conversely, Dowthwaite and Parikh found that the timing of TEP had no effect on pharyngocutaneous fistula formation [10,11]. A subsequent large meta-analysis found that this risk is most noticeable with combined chemoradiotherapy over simply radiotherapy [12]. In the growing population of twice-radiated patients, half of them in Clancy’s group had ultimate removal of their voice prosthesis with removal occurring at a median of 24.9 months after placement. Reasons for prosthesis removal included widening tracheoesophageal fistula, local recurrence, and dysphagia/esophageal stenosis. Nearly one-third of these patients required surgical intervention for the closure of a widening fistula [13].

Interestingly, although pharyngocutaneous fistula is probably the most feared complication from TEP [14], the most common reason for total TEP failure is dissatisfaction with voice (26.3%). Less commonly, TEPs fail due to leakage (17.9%), inadequate patient motivation (14.7%), comorbidities (14.2%), stoma problems (11.6%), and abandonment of TEP after dislodgement (10.6%) [15,16]. There are several described surgical techniques for closure of tracheoesophageal fistulae following TEP if TEP is no longer desired, including local, regional, and free flaps [17,18,19,20].

While TEP use has been associated with greater quality of life in laryngectomy patients [21], TEP patients have been documented to flourish most when involved in multidisciplinary care that integrates speech therapy, oncologic surveillance, and psychosocial services [22]. Factors that affect patients’ satisfaction with their TEP can be unexpected. For example, Galli found that distance from a patient’s home to their provider’s facility greatly impacted patients’ satisfaction with their TEP. Twenty percent of his patients with successful TEP voicing would not undergo TEP placement again if they had the chance to make their decision again, mostly due to the distance traveled for care [23]. Patients must be able to make routine visits to their institution, as normal changes in the tracheoesophageal tract can dictate frequent resizing of the tract, which can change in length and diameter over time [24]. Swelling generated by the creation of the fistula, surgery, and radiation gradually decreases. This necessitates repeated measurements of the length and diameter of the TEP tract by a speech therapist, who can select an appropriately sized prosthesis [25,26].

Since first described by Singer and Blom in 1980, the TEP has been often considered the gold standard for voice restoration following laryngectomy [3]. A variety of methods of secondary prosthesis placement have been described. The earliest methods relied on rigid esophagoscopy for TEP placement, and rigid esophagoscopy is still a commonly used technique for TEP placement. However, patients with difficult cervical anatomy due to kyphosis, limited neck range of motion, neck scarring, bulky flaps, or severe radiation related changes may be at increased risk of esophageal perforation and mediastinitis, cervical spine injury, dental injury, and unsuccessful TEP with rigid techniques [27,28].

Alternative methods of secondary TEP placement have been described, including flexible esophagoscopy with insufflation during general anesthesia, awake transnasal flexible esophagoscopy, and endoscopy through an endotracheal tube (ETT) placed into the esophagus [29,30,31,32]. These methods, however, do not provide protection to the posterior tracheal wall. Methods attempting to combine a flexible approach with the protection of the posterior esophageal have also been described, including puncture through a gastric lavage while in the esophagus [33] and puncture through the small fenestration hole of a rigid suction catheter [34]. 

In this paper, we describe a method of secondary TEP placement using a fenestrated chest tube for esophageal access and stenting which allows for minimal neck extension, optimal protection of the posterior esophageal wall and oral structures, and even allows for a transnasal approach in the patient with trismus.

## 2. Methods

This project was approved through expedited review by WCG Institutional Review Board. It was granted approval under protocol number: 2022-002. The Protocol Title was “A retrospective review of clinical outcomes from the West Cancer Center and Research Institute: An Early Adopting Community Cancer Hospital.” On 14 April 2022, WCG IRB approved a request for a waiver of authorization for use and disclosure of protected health information (PHI) for the above-referenced research. 

The patient is typically placed under general anesthesia, but this technique can be performed in an endoscopy suite with adequate local anesthesia and sedation. A hockey-stick ultrasound probe placed through the tracheostoma can be used to measure the thickness of the tracheoesophageal parti-wall. This allows the selection of the correct-length TEP in advance [35,36]. Maloney dilators are optionally used to serially dilate the esophageal lumen to 40 Fr in order to accommodate a 36 French chest tube with ease.

**Figure 1:**
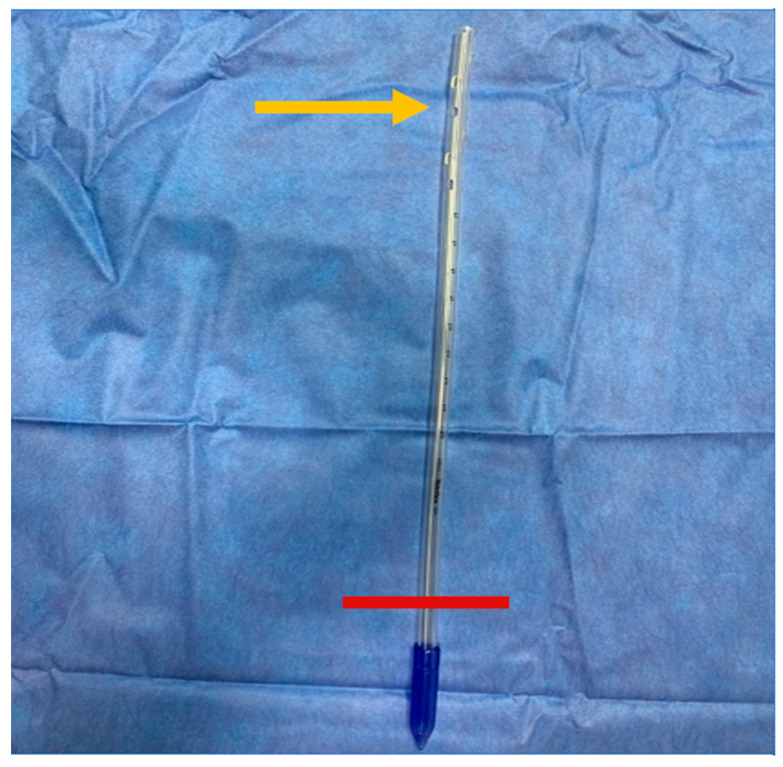
The tapered end of the chest tube is cut (red line) and the fenestrated end (orange arrow) is inserted into the mouth.

**Figure 2:**
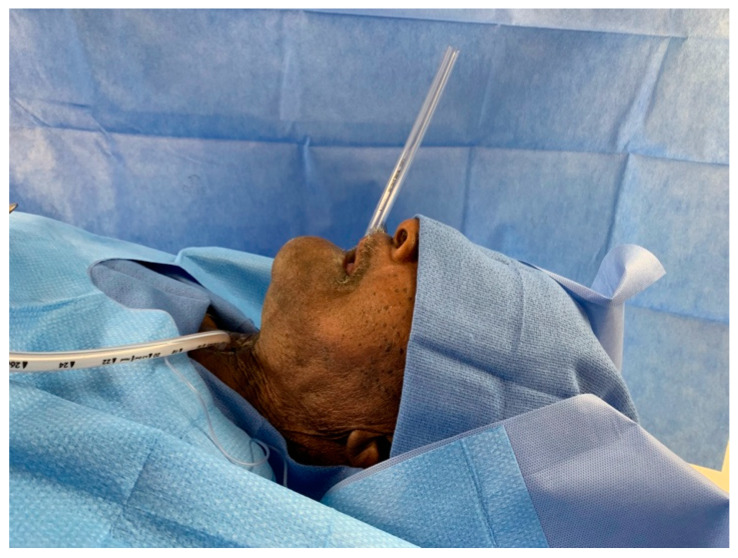
The fenestrated end is advanced into the esophagus. The cut tapered end is external and provides an opening for scope insertion. As seen here, the chest tube can be placed with limited neck extension. The length protruding from the mouth need only be long enough to maintain control of it while passing the flexible endoscope.

A 36 French chest tube is placed through the patient’s mouth in a retrograde fashion, i.e., passing the end with the fenestrations (not the tapered end) into the neopharynx and esophagus. The tube is advanced until one of the fenestrations of the chest tube is palpable behind the planned TEP puncture site in the trachea. The tapered end of the chest tube is trimmed so that 7–10 cm are protruding from the patient’s mouth (Figure 1 and Figure 2).

A flexible bronchoscope or small diameter flexible esophagoscope is used. In our practice, we use an Ambu aScope^TM^ 4 Broncho Regular (OD 5.0 mm/0.20″, ID 2.2 mm/0.09″) flexible bronchoscope. This has a small video screen and is on a portable tower.

**Figure 3:**
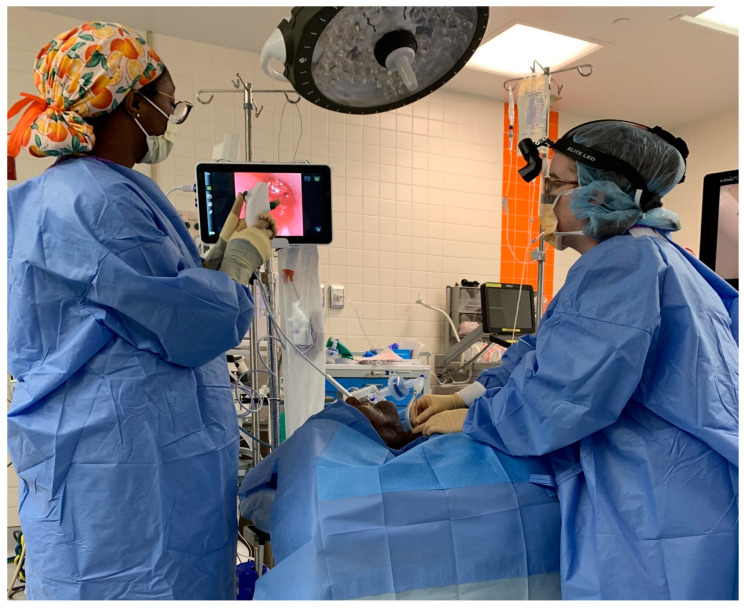
The surgeon and endoscopist work together to place the trocar through the fenestration in the anterior wall of the chest tube under endoscopic view.

**Figure 4:**
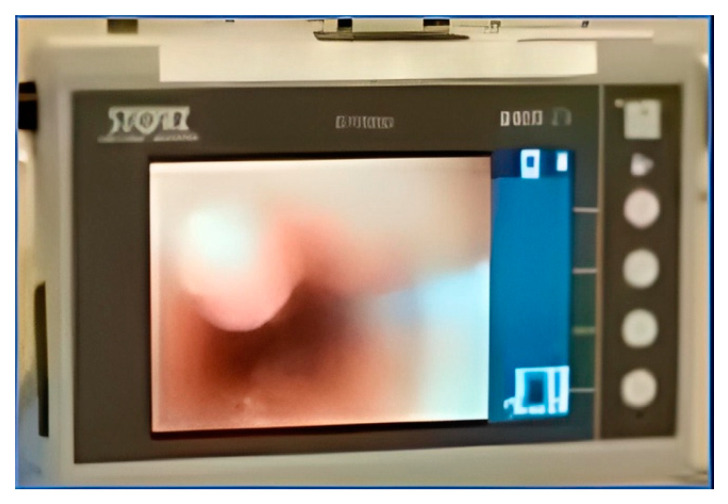
A fenestration in the chest tube is advanced until it is underlying the desired puncture site. Appropriate positioning can be confirmed by palpating in the fenestration with a hemostat.

**Figure 5:**
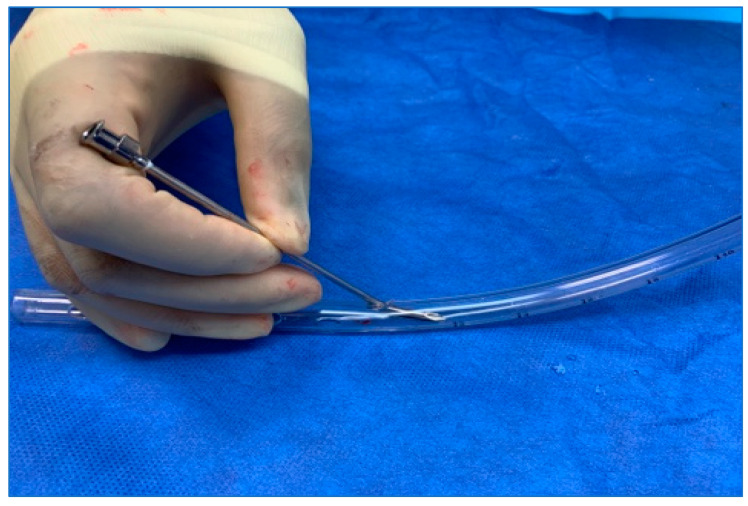
Demonstration of the trocar insertion technique. The chest tube is the ideal adjunct for trocar insertion as it is flexible, provides an opening for insertion, and protects the esophagus posteriorly.

**Figure 6:**
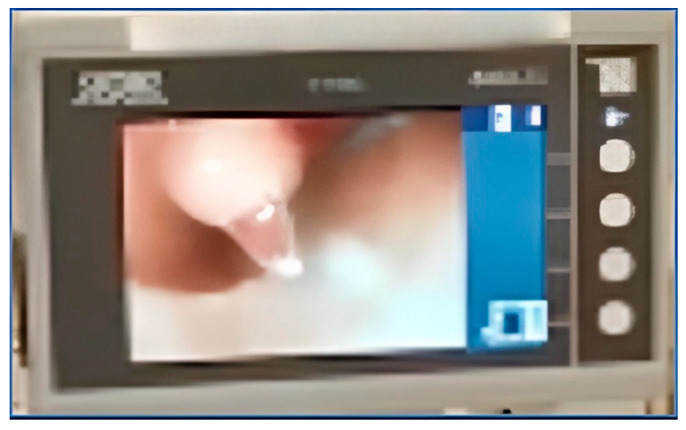
Insertion of trocar under endoscopic visualization. The chest tube protects the posterior esophageal wall from injury.

**Figure 7:**
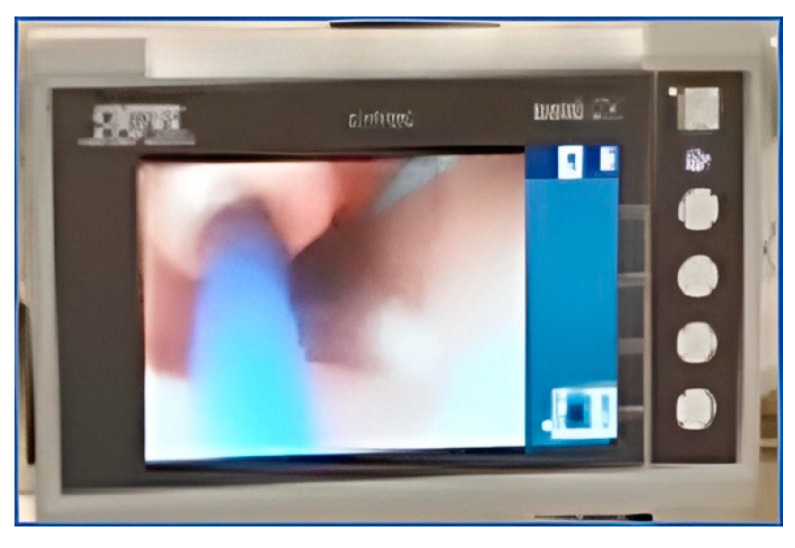
The guidewire can be advanced cranially under endoscopic visualization.

The flexible scope is placed into the lumen of the chest tube. Usually, lubrication is not necessary. The scope is advanced to the esophagus just behind the TEP puncture site. This is confirmed by viewing the light through the tracheal wall, and by palpating the tracheal mucosa at the puncture site with the tip of a hemostat so that the movement can be viewed through the chest tube fenestration via the esophagoscope (Figure 3 and Figure 4). As the trocar from a TEP puncture kit is placed through the posterior tracheal wall into the esophageal lumen, the endoscopist watches the trocar being pushed through the esophagus and into the lumen of the chest tube through one of the fenestrations (Figure 5). The trocar can safely be advanced cranially, with the chest tube protecting the posterior esophageal wall from injury (Figure 6). A guidewire is placed through the trocar under endoscopic vision and advanced until it emerges from the end of the chest tube protruding from the mouth (Figure 7). The tracheal end of the guidewire is held securely as the trocar is removed from the trachea. The flexible scope and chest tube are withdrawn from the mouth, keeping control of the oral end of the guidewire as well. The prosthesis is then locked onto the guidewire and pulled through the pharynx, esophagus, and trachea until the flange emerges to rest against the tracheal mucosa.

An instructional video demonstration has been made available for further clarity (https://www.youtube.com/watch?v=NMXy-8grbDs).

## 3. Results

We have utilized this method using a chest tube in dozens of total laryngectomy patients over the years (Table 1). The average age of our patients was 62 years. The average time from laryngectomy to placement of secondary TEP was 197 days. Most of our patients were male. The majority of patients had undergone a previous attempt at TEP with another technique (74%), and 92% of our patients had received prior radiation.

All patients underwent successful placement of a TEP under general anesthesia through a chest tube and with flexible endoscopic guidance. Some of the patients had undergone previous failed attempts at TEP placement using rigid transoral esophagoscopy, or had been deemed impossible to rigidly scope and thus obtaining a TEP was unfeasible. No patients suffered pharyngoesophageal injury or perforation, there were no dental injuries, false tracheoesophageal passage was avoided, and all patients left the operating room with a voice prosthesis in place.

## 4. Discussion

Since 1980, the placement of secondary TEP has been described with several modifications. Initially, this was described using a rigid esophagoscope under general anesthesia. In patients with trismus and restricted neck extension in which a rigid esophagoscope could not be placed, flexible transnasal esophagoscopy (TNE) was used.

Glazer described performing TEP placement in the office using topical anesthesia, TNE, and a central venous catheter kit. After topical anesthesia was administered, this group used an 18 g introducer needle, guidewire, and dilator from the central venous catheter kit. This method was found to have a much lower cost than procedures in the operating room [37]. Unfortunately, patients that could not tolerate TNE were not candidates for this technique.

Tkaczuk also investigated ways to perform TEP in patients with esophageal stricture, limited neck extension, and soft-tissue fibrosis. He realized the advantages of the flexible esophagoscope over the rigid esophagoscope including magnification, superior clarity, illumination, insertion via the nose or mouth, and a working channel to permit interventions such as balloon dilation and biopsy. He described TNE using a collapsable mesh device that was deployed through the working port of the flexible esophagoscope. The mesh was expanded when deployed and applied radial force to distend the esophagus and maintain patency while the needle was placed transluminally into the esophagus. This method was successful in cadavers [38], but the TNE technique can be limited by patient discomfort and visualization problems. Some patients have difficulty tolerating large instruments in their nasal cavity and having a puncture placed in their tracheoesophageal wall while awake in the office. Bleeding at the level of the distal scope can also be a hindrance.

Ricci developed an in-office technique for TEP placement in patients with comorbidities that prevented them from being placed under general anesthesia. His technique of retrograde voice prosthesis placement allowed the immediate placement of the prosthesis and the avoidance of the use of dilators. Unfortunately, his technique required flexible transoral esophagoscopy as well as intravenous sedation and esophageal insufflation in some patients. A gastroenterologist was also present. There was no protection to the posterior wall of the esophagus, and there was increased risk in patients who had scarring from a previous TEP [39].

Wu has described the exchange of a pre-existing TEP in the office with a retrograde technique, but not a new TEP placement. He has used his technique using a red rubber catheter in challenging patients with flap reconstruction, history of esophageal strictures requiring dilation, and history of stoma stenosis. He admits that this technique cannot be used if the pre-existing prosthesis cannot be oriented in a superiorly directed trajectory [40]. Historically, voice prosthesis exchange has involved removing the existing prosthesis followed by anterograde placement of the new prosthesis, either with a gel cap or insertion device [41,42].

General anesthesia has proven helpful in these already challenging patients. Maniglia described using an endotracheal tube as an esophageal stent in 1982. He cut a window in the endotracheal tube for the trocar and guidewire to be placed [43]. Sharma and Padhya described placing the trocar through the distal bevel of the size 9 endotracheal tube [29]. Abdul-Aziz felt that these alterations increased the difficulty of the procedure and therefore the danger to the patient. In 2018, he described passing the endotracheal tube through the mouth and into the esophagus to the level of the tracheal stoma. The curved needle and associated sheath were advanced through the tracheoesophageal wall to pierce the anterior aspect of the endotracheal tube under direct fiberoptic visualization. This can still require significant force to pierce the stiff endotracheal tube, risking injury to the posterior esophageal wall. The endotracheal tube is slowly withdrawn several centimeters, pulling the needle and sheath further into the esophageal lumen [44].

In our technique, we use a chest tube instead of an endotracheal tube. The chest tube is inserted distal to the TEP site, and the trocar is inserted into an existing fenestration (Figure 3, Figure 4, Figure 5 and Figure 6). There are multiple pre-existing oval fenestrations in the wall of the chest tube. Therefore, no force is needed to advance the trocar into the chest tube. After passing through the fenestration, the guidewire easily advances through the lumen of the chest tube. The chest tube can remain in place as the guidewire is advanced superiorly towards the oral cavity, rather than progressively pulling it out as other methods require.

This method provides improved protection of the posterior wall of the esophagus over other techniques with the benefits of a flexible technique that requires minimal neck extension. This technique also excels in that it allows for excellent transillumination and tactile feedback at the desired puncture site without compromising endoluminal visualization. This method has proven successful in patients with severe trismus with no mouth opening. The same technique of using a chest tube can be utilized through the nose after adequate decongestion and dilation.

The described method was initially used by our group in patients deemed poor candidates for standard rigid esophagoscope placement procedures. Due to the ease of placement and excellent visualization, this method has become the authors’ preferred method for secondary TEP placement in all patients, provided that a qualified assistant is available. We advocate for this method of TEP placement as the method is simple and effective, and all materials used are readily available at any busy surgical center. To the authors’ knowledge, no complications or adverse effects specific to this technique have been observed.

Scherl recommends placing the TEP during the primary TL surgery or at least before scheduled radiotherapy [45]. Even though 94% of our patients had radiation prior to TEP placement, we did not have complications.

None of our patients had postoperative complications such as aspiration, dislodgement, dysphagia, poor voice, pharyngoesophageal injury or perforation, dental injuries, or false tracheoesophageal passage. This is surprising, as complications with secondary TEP are common (65.2%). The most frequent problem reported with secondary TEP is salivary leakage (50.0%) [45].

The length of time from TL to TEP was considerable for our patients because most of these patients had prior attempts at TEP with another technique. Some of these patients have other regular providers and were referred to us after cancer treatment and laryngectomy was performed by other physicians. The recommended timeframe for secondary placement after radiotherapy is broad, as some patients have delayed healing due to microvascular tissue damage caused by irradiation [28,46,47] or have overall poor status and need time to recover from radiation. Neumann and Schultz-Coulon have recommended at least 12 months after radiotherapy before placing TEPs [46].

It is very challenging to compare results of our patients with other techniques, because most published techniques do not include detailed information regarding patients background or outcomes. These are mostly descriptive techniques in a few patients or cadaveric specimens and do not report patient demographics, previous treatment, or long-term outcomes. Some of the studies describe the techniques in cadavers, so outcome comparisons are not possible.

This method does have several limitations. Although minimal, our technique may still require a small amount of neck extension. The trocar must be able to be angled parallel to the chest wall and a small amount of neck extension may be required in select patients in order to insert the trocar into the tracheal stoma. Many patients still report some neck pain following the procedure, and these patients typically have kyphosis and/or a history of radiation. A large-diameter chest tube is utilized by the authors, as smaller chest tubes have been observed to have excess motion during trocar placement, and are not large enough to accommodate readily available bronchoscopes or smaller esophagoscopes. If the patient’s esophagus cannot be safely dilated to 40 Fr prior to chest tube placement, the procedure may need to be aborted. Ideally, the patient will have been screened for presence of strictures while determining their candidacy for TEP utilization. Finally, this procedure, like all other TEP placement techniques thus described, requires a surgeon and an experienced assistant.

## 5. Conclusions

We describe a method of TEP placement which we believe to be simple, efficient, safe, and reliable with supplies readily available at any busy surgical center. Using a chest tube for esophageal stenting and trocar placement allows for minimal neck extension, no need for oral opening, optimal transillumination and tactile feedback at the puncture site, protection of the posterior esophageal wall, and excellent endoluminal visualization with endoscopic instruments.

## Figures and Tables

**Table 1:** Characteristics of patients undergoing chest tube placement of secondary TEP.

Patient Characteristics (N = 76)	
Average age	62 years
Average time from TL to TEP	197 days
Sex (M:F)	3.1:1.2
Prior attempt with another technique	74% (n = 56)
Prior XRT	92% (n = 70)

## Data Availability

Data are contained within the article.

## Abbreviations

Total laryngectomy (TL); tracheoesophageal puncture (TEP), endotracheal tube (ETT), flexible transnasal esophagoscopy (TNE).
